# *N*-acetyltransferase AAC(3)-I confers gentamicin resistance to *Phytophthora palmivora* and *Phytophthora infestans*

**DOI:** 10.1186/s12866-019-1642-0

**Published:** 2019-11-27

**Authors:** Edouard Evangelisti, Temur Yunusov, Liron Shenhav, Sebastian Schornack

**Affiliations:** Sainsbury Laboratory Cambridge University (SLCU), Cambridge, UK

**Keywords:** Oomycete, Counter-selection, Aminoglycoside, N-acetyltransferase, Specificity

## Abstract

**Background:**

Oomycetes are pathogens of mammals, fish, insects and plants, and the potato late blight agent *Phytophthora infestans* and the oil palm and cocoa infecting pathogen *Phytophthora palmivora* cause economically impacting diseases on a wide range of crop plants. Increasing genomic and transcriptomic resources and recent advances in oomycete biology demand new strategies for genetic modification of oomycetes. Most oomycete transformation procedures rely on geneticin-based selection of transgenic strains.

**Results:**

We established N-acetyltransferase AAC(3)-I as a gentamicin-based selectable marker for oomycete transformation without interference with existing geneticin resistance. Strains carrying gentamicin resistance are fully infectious in plants. We further demonstrate the usefulness of this new antibiotic selection to super-transform well-characterized, already fluorescently-labelled *P. palmivora* strains and provide a comprehensive protocol for maintenance and zoospore electro-transformation of *Phytophthora* strains to aid in plant-pathogen research.

**Conclusions:**

N-acetyltransferase AAC(3)-I is functional in *Phytophthora* oomycetes. In addition, the substrate specificity of the AAC(3)-I enzyme allows for re-transformation of geneticin-resistant strains. Our findings and resources widen the possibilities to study oomycete cell biology and plant-oomycete interactions.

## Background

Oomycetes are filamentous microbes that grow as saprotrophs or as pathogens of a wide range of hosts from various lineages such as insects, fish, mammals including humans, and plants [1, 2]. Diseases caused by members of the plant-pathogenic oomycete genus *Phytophthora* have a strong economic footprint and therefore have received extensive attention over the past decades. For instance, late blight of tomato and potato due to infection with *Phytophthora infestans*, a member of clade 1 [3, 4], is responsible for billion-dollar losses yearly [5]. Similarly, the broad-host-range tropical species *Phytophthora palmivora* from clade 4 [3, 4] triggers disease on economically relevant crops including cocoa, mango, papaya, rubber tree, oil palm and many *Citrus* species [6, 7]. In addition, some *Phytophthora* are detrimental to natural ecosystems. For example, *Phytophthora ramorum* is threatening tanoak and other oak species in California and Oregon [8], and *Phytophthora cinnamomi* causes disease on multiple trees across the world, such as chestnut, oak, *Eucalyptus* and *Banksia* [9]. Most of these species are spreading beyond their original geographic area due to international trade and climate change [2, 10].

*Phytophthora* infection relies on the production of flagellate zoospores that reach host tissues by chemo- and electrotaxis [11]. Adhering zoospores encyst and germinate. Then, the germ tube rapidly differentiates into an appressorium-like structure to enable host penetration [12]. Following is a biotrophic stage characterized by oomycete hyphae growing extracellularly with no damage to host cells, and differentiating digit-like structures termed haustoria to deliver effectors [11, 13]. Biotrophy is then followed by a more detrimental stage, termed necrotrophy, causing death to host tissues. The oomycete completes its lifecycle by differentiating sporangia which produce new zoospores, further spreading the infection [14, 15].

Genomic [16–18], transcriptomic [15, 19], proteomic and metabolomic [20, 21] resources have been obtained to help deciphering the molecular basis of oomycete virulence. Genetic manipulation of oomycetes has gained increasing interest with the study of their molecular weaponry and the modalities of host tissue colonization [22–26]. At least four methods have been successfully applied to transform oomycetes: liposome-mediated protoplast transformation [27], microprojectile bombardment [28], *Agrobacterium*-mediated transformation [29, 30] and electroporation [31]. By contrast, only a handful of vectors are commonly used for delivery and genomic integration of transgenes in oomycetes. Most of them carry the *Bremia lactucae* Ham34 constitutive promoter [32] and native promoters are rarely used [25, 33]. The selection of oomycete transformants relies on the aminoglycoside antibiotics geneticin (G418) or hygromycin B [27].

Aminoglycosides antibiotics are synthesized by bacteria from the genera *Streptomyces* and *Micromonospora* [34]. They bind to the prokaryotic ribosomal decoding site, thereby reducing the fidelity of protein synthesis and ultimately killing susceptible bacteria [35]. The isolation of aminoglycoside-inactivating enzymes has widened their usage in basic research to assist with bacterial transformation. In addition, some antibiotic/enzyme combinations have been successfully used for the selection of transfected eukaryotic cells [36, 37]. Enzymatic inactivation of aminoglycosides can be achieved through acetylation, adenylylation, and phosphorylation [38] and several enzyme classes exist for each of these modifications. For instance, four classes of *N*-acetyltransferases inactivate aminoglycosides by acetylation of the 1-, 3-, 2′- and 6′-amino groups, respectively, conferring partially overlapping aminoglycoside resistance profiles [38].

Here we demonstrate that the aminoglycoside gentamicin arrests *P. palmivora* and *P. infestans* growth in vitro. The *N-acetyltransferase* AAC(3)-I confers gentamicin resistance, but retains geneticin (G418) susceptibility in *P. palmivora.* We generated Gateway compatible pTOR vectors for gentamicin-based selection to super-transform G418-resistant *P. palmivora.* This enabled fluorescent labelling of multiple cellular compartments and structures. Our findings and materials extend antibiotic selection as well as genetic manipulation possibilities for oomycetes.

## Results

### Gentamicin inhibits *P. palmivora* and *P. infestans* growth in vitro

**Fig. 1:**
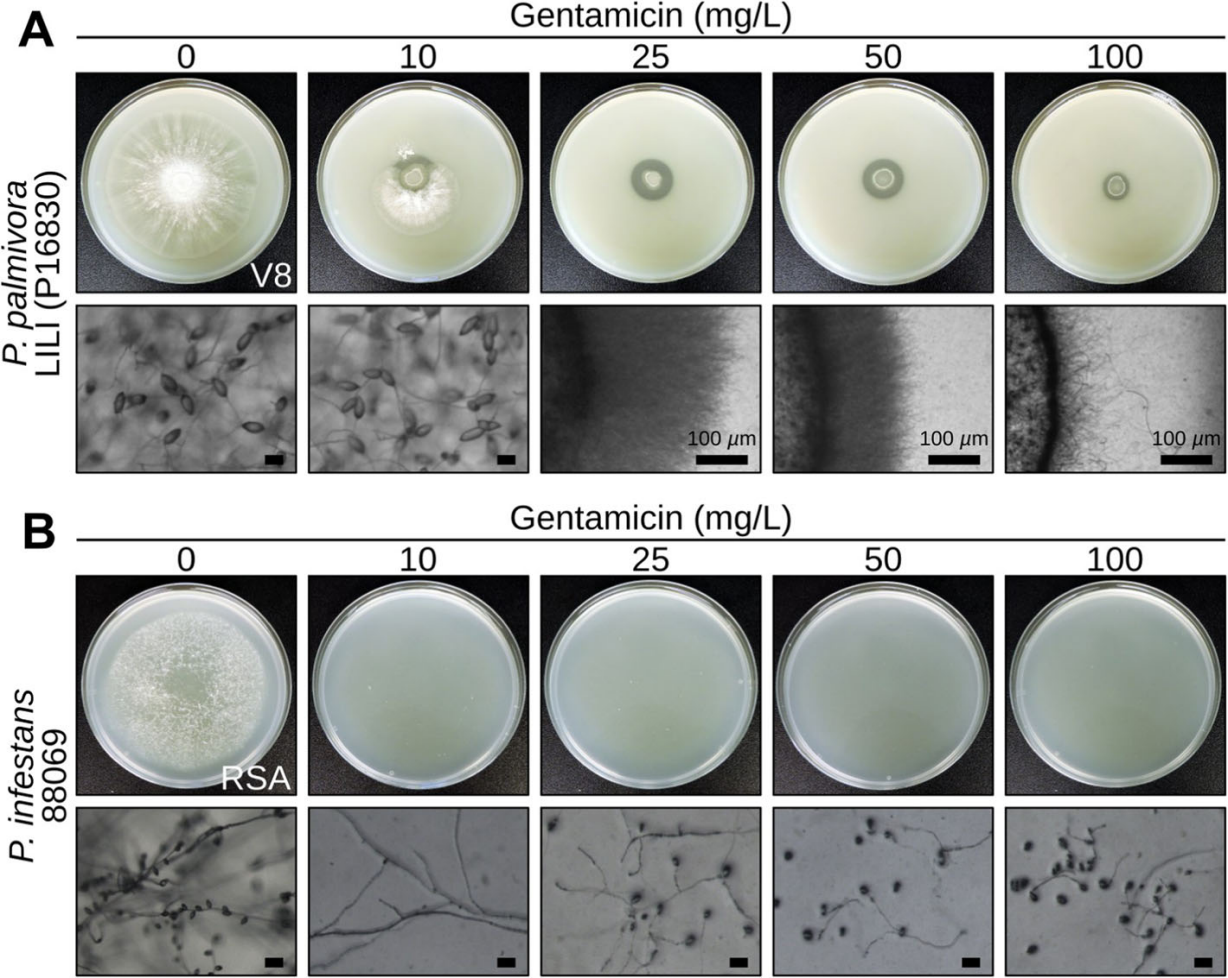
Gentamicin impairs growth of wild-type and G418-resistant *Phytophthora palmivora* and *Phytophthora infestans* strains in vitro. **a-b)** Representative pictures of 5-day-old *P. palmivora* LILI (accession P16830) grown on V8 (**a**) or 10-day-old *P. infestans* isolate 88,069 grown on RSA (**b**). Plates were supplemented with 0, 10, 25, 50 or 100 mg/L gentamicin. Scale bar is 30 μm

To expand the possibilities for antibiotic selection after transformation we surveyed *P. infestans* and *P. palmivora* for their susceptibility to carbenicillin, chloramphenicol, cefotaxime, gentamicin, rifampicin, spectinomycin and tetracycline. While most antibiotics were not effective in limiting mycelial growth **(**Additional file 1: Figure S1**)**, *P. palmivora* and *P. infestans* were both susceptible to gentamicin **(**Fig. 1**)**. A concentration of 10 mg/L gentamicin **(**Fig. 1a**)** limited *P. infestans* hyphal growth and inhibited sporangia formation. By contrast, some *P. palmivora* colonies were still able to grow and produce sporangia at this concentration **(**Fig. 1b**)**. At 100 mg/L gentamicin, development of both oomycete species was fully arrested at the germinating cyst stage **(**Fig. 1a-b**)**. Thus, gentamicin-100 is a robust and reproducible inhibitor of mycelial growth on V8 and RSA agar growth media.

### Gentamicin is a reliable selectable marker for *P. palmivora*

**Fig. 2:**
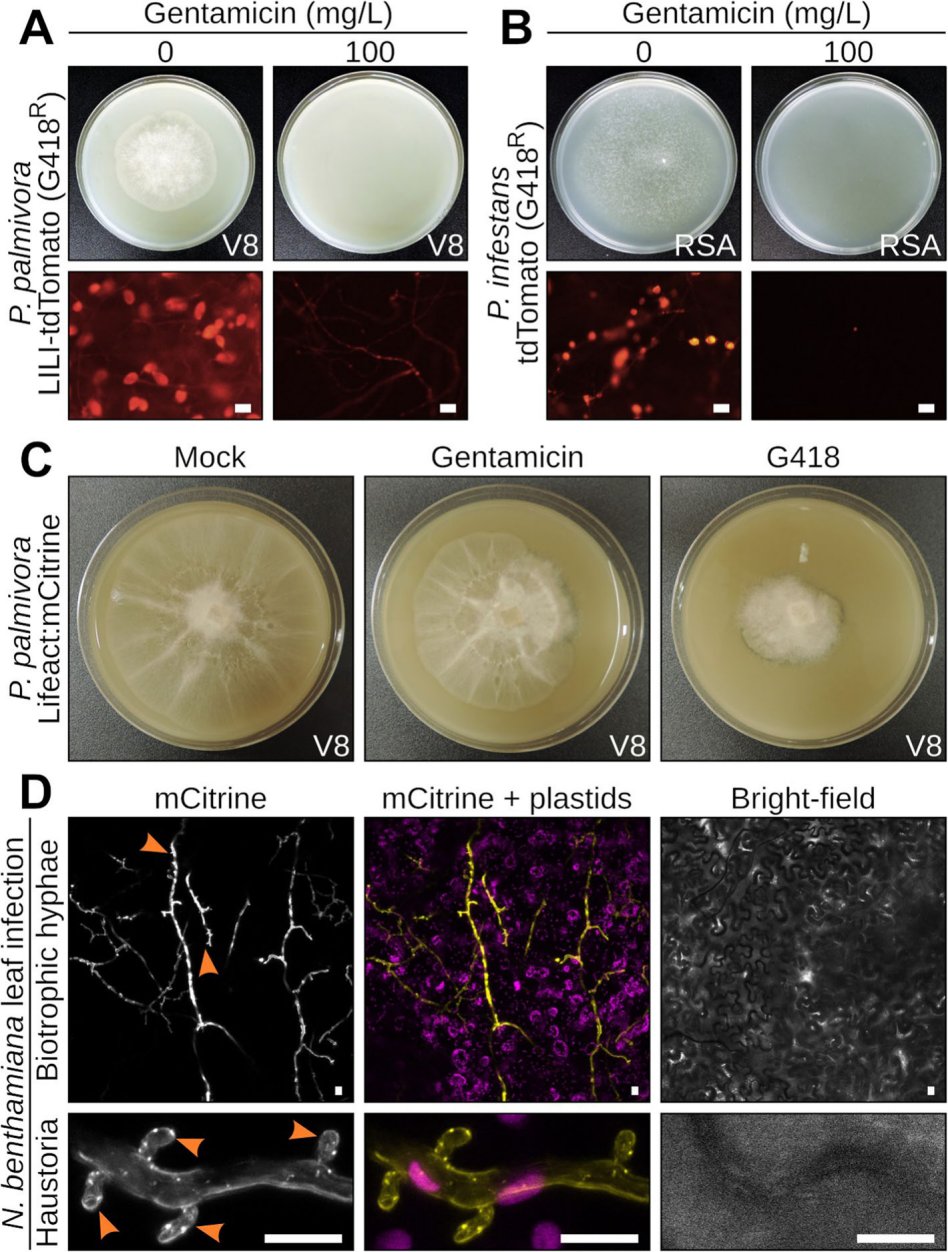
Gentamicin is a reliable selectable marker for *Phytophthora* (**a-b)** Representative pictures of 5-day-old transgenic *P. palmivora* LILI (**c**) or 10-day-old transgenic *P. infestans* (**d**) carrying a construct for constitutive expression of the tdTomato fluorescent protein. Plates were grown on V8 or RSA, respectively, and supplemented or not with 100 mg/L gentamicin. **c** Representative pictures of 5-day-old transgenic *P. palmivora* LILI transformed with a gentamicin-based pTOR vector, grown on V8 plates containing either no antibiotic (left), 100 mg/L gentamicin (middle) or 100 mg/L geneticin (G418, right). **d** Representative pictures of infectious hyphae from a gentamicin-resistant *P. palmivora* strain expressing a constitutive Lifeact:mCitrine reporter, 24 h after infection of a *Nicotiana benthamiana* leaf. Arrowheads indicate haustoria. Scale bar is 10 μm

To determine whether gentamicin-based selection could be used on G418-resistant *Phytophthora*, we assessed growth of transgenic *P. palmivora* and *P. infestans* strains carrying the *neomycin phosphotransferase* (*nptII*) resistance gene on vegetable V8 juice agar plates containing 100 mg/L gentamicin **(**Fig. 2a-b**)**. We found that the growth of transgenic *P. infestans* **(**Fig. 2a**)** and *P. palmivora* **(**Fig. 2b**)** strains expressing tdTomato was impaired on selective plates containing gentamicin. Thus, the *nptII* gene does not confer resistance to gentamicin.

To determine whether gentamicin can be used as a selectable marker for *P. palmivora*, we generated a set of pTOR-Gateway vectors carrying the *aminoglycoside 3-N-acetyltransferase I* (*aac(3)-I* or *aacC1*) gene from *Pseudomonas aeruginosa* [39] as a replacement for *nptII* **(**Additional file 1: Table S1**)**. Using an improved electroporation approach (Supporting Protocol), we transformed the wild-type *P. palmivora* strain LILI with a pTORGm43GW vector carrying a construct for constitutive expression of an actin-labelling Lifeact:mCitrine reporter under control of the Ham34 promoter. Transformants grew on V8 medium containing gentamicin **(**Fig. 2c**)**, suggesting that Hsp70*pro*-driven *aacC1* expression efficiently detoxified gentamicin. Furthermore, the growth of gentamicin-resistant *P. palmivora* strains on V8 plates supplemented with G418 was attenuated **(**Fig. 2c**)**, confirming that *aacC1* does not confer cross-resistance to G418. In addition, gentamicin-resistant *P. palmivora* strains were able to infect *Nicotiana benthamiana* leaves and formed intracellular haustoria **(**Fig. 2d**)**, suggesting that expression of the AAC (3)-I enzyme does not impair the virulence of these strains. Taken together, gentamicin is a reliable selectable marker for *P. palmivora*.

### Gentamicin-based vectors for super-transformation of G418-resistant *Phytophthora* strains

**Fig. 3:**
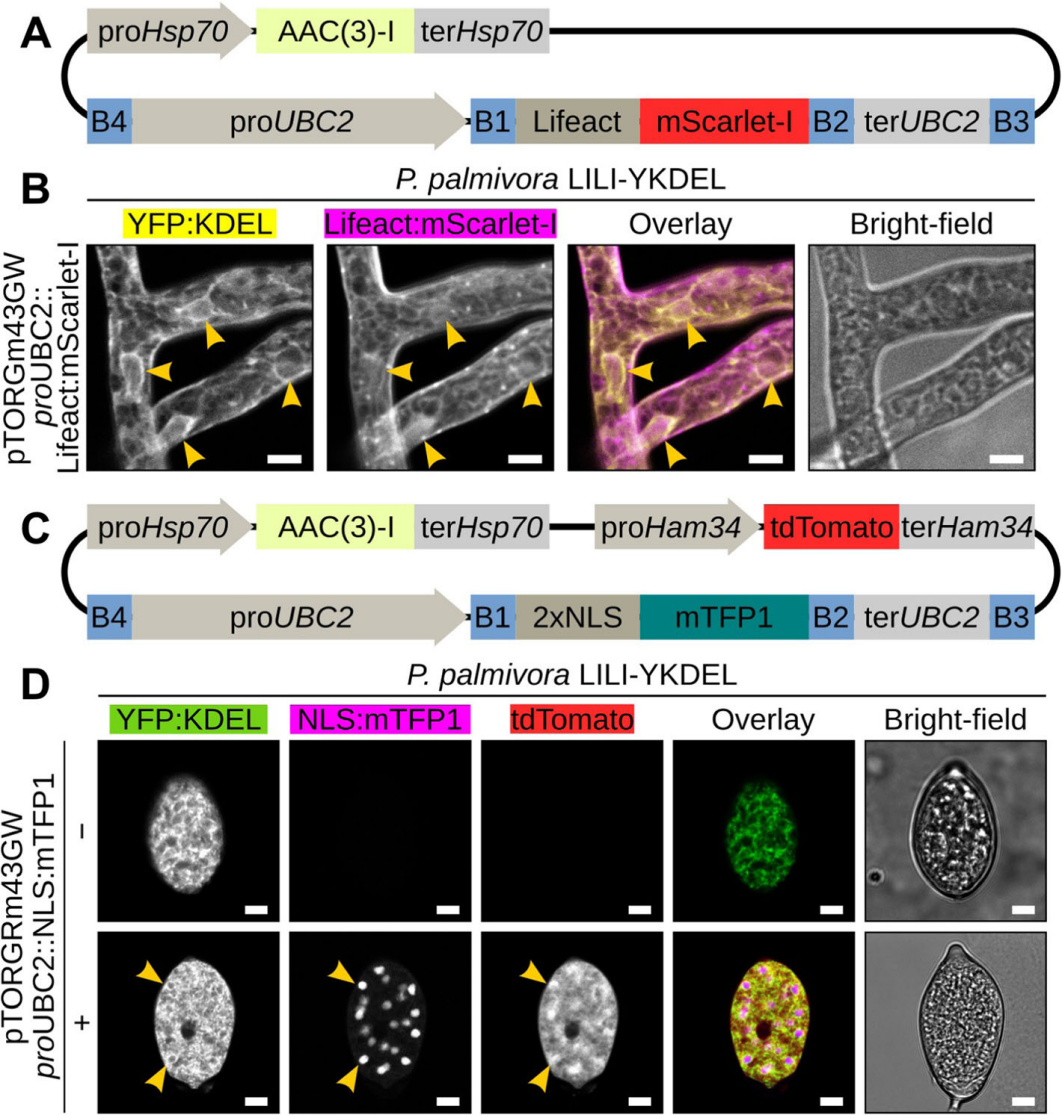
Gentamicin-based pTOR vectors enable super-transformation of G418-resistant *P. palmivora* strains. **a** A transgenic *P. palmivora* LILI-YKDEL strain was transformed with a gentamicin-based vector carrying a construct for constitutive expression of a Lifeact:mScarlet-I reporter. **b** Representative pictures of hyphae grown axenically on V8 plate containing 100 mg/L gentamicin and G418. **c** The same strain was transformed with a gentamicin-based vector carrying a construct for constitutive expression of a nuclear-localized mTFP1 in addition to a cytoplasmic tdTomato marker. **d** Representative pictures of hyphae grown axenically on V8 plate containing 100 mg/L gentamicin and G418. Arrowheads indicate nuclei. Scale bar is 10 μm

Next, we assessed the possibility to perform dual selection using both G418 and gentamicin **(**Fig. 3**)**. To that end, we transformed the G418-resistant *P. palmivora* LILI-YKDEL strain [15, 40] with vectors carrying the *aacC1* gene in addition to a construct for constitutive expression of either an actin-labelling Lifeact:mScarlet-I fluorescent reporter **(**Fig. 3a-b**)** or a cytoplasmic tdTomato and a nuclear-localized mTFP1 fluorescent protein **(**Fig. 3c-d**)**. All regenerated transformants were able to grow on V8 medium containing both G418 and gentamicin and expressed the different reporter genes in their respective subhyphal compartments **(**Fig. 3b, d**)**. Hence, pTOR-Gateway vectors carrying a gentamicin resistance cassette allow for super-transformation of G418-resistant transgenic *P. palmivora* strains.

## Discussion

Here we document that gentamicin is a robust selectable marker for *P. palmivora* that can be used for transformation of wild-type and G418-resistant strains. Many aminoglycosides are primarily used as bactericidal antibiotics. They inhibit protein synthesis by binding to the A-site on the 16S ribosomal RNA of the 30S bacterial ribosome [41, 42]. Besides its activity in prokaryotes, gentamicin selection was used as an efficient selectable marker in eukaryotic plants such as *Petunia hybrida* [43] and *Nicotiana tabacum* [44]. Efficient gentamicin-based selection was also reported for *Arabidopsis thaliana* [43] and, more recently, for the liverwort *Marchantia polymorpha* [45], although no mechanism of action has been proposed so far. In addition, a bifunctional enzyme conferring resistance to both gentamicin and tobramycin was used for selection of *N. tabacum* transplastomic lines [46], taking benefit of the prokaryotic translational apparatus of chloroplasts [47]. The low affinity of aminoglycosides for eukaryotic ribosomes is due to differences at two key nucleotides of the ribosomal RNA that occupy the ribosome decoding centre [48, 49]. However, a few aminoglycosides bind to eukaryotic ribosomes are thus are used in nonsense suppression therapy to suppress translation termination at in-frame premature termination codons [50]. Whether gentamicin binds to oomycete ribosomes or mitochondrial ribosomes (mitoribosomes) remain to be determined. Indeed, studies of hybrid bacterial ribosomes containing a decoding site mimicking the human mitochondrial 12S rRNA showed altered protein translation fidelity in the presence of aminoglycosides, suggesting aminoglycosides can interfere with mitoribosomes function [51]. In addition, gentamicin may interfere with other key metabolic processes. For instance, some reports suggest that gentamicin may suppress the ADP ribosylation factor (ARF)-dependent protein trafficking [52].

We found that the nptII selectable marker expressed by G418-resistant *P. palmivora* strains does not confer resistance to gentamicin, and that growth of *P. palmivora* strains carrying the aacC1 selectable marker was arrested on V8 plates containing G418, but not gentamicin. Our data are consistent with the specificity of these aminoglycoside processing enzymes. The gene *aacC1* [39] used in this study encodes the AMINOGLYCOSIDE 3-N-ACETYLTRANSFERASE I (AAC(3)-I), which has narrow substrate specificity and can only acetylate gentamicin, astromicin and sisomicin [38]. The *nptII* gene derived from the Tn5 transposon encodes the AMINOGLYCOSIDE 3′-PHOSPHOTRANSFERASE II (APH(3′)-II) which can phosphorylate kanamycin, G418 and gentamicin B, but not members of the gentamicin C complex [38]. Gentamicin C constitutes 80% of the gentamicin sulphate preparations [53] and has more potent antimicrobial activity than the remaining 20% of so-called minor components (mostly gentamicins A, B and X) [54]. Considering substrate specificities of the APH(3′)-II and AAC(3)-I enzymes and composition of the gentamicin antibiotic was crucial for the success of double selection approaches in this study.

Under natural conditions, fungi and oomycetes are often associated with a broad range of bacteria and inter-kingdom communication has been shown [55–57]. While it cannot be excluded that bacterial associations with *P. palmivora* may provide host range or environmental benefits, our data suggests that *P. palmivora* strains obtained after several rounds of cultivation on V8 medium containing a mixture of bactericidal and bacteriostatic antibiotics are still capable of readily infecting *N. benthamiana*. Future work will investigate whether such isolates perform worse on less compatible hosts and whether they are indeed axenic or still have antibiotic resistant bacteria associated with them.

## Conclusions

In this study we highlight the usefulness of gentamicin-based selectable marker in oomycetes. We provide evidence for the functionality of the N-acetyltransferase AAC(3)-I in *Phytophthora*, and demonstrate that it enables super-transformation of well-characterized, G418-resistant strains. We take advantage of these findings to develop a versatile toolbox of gentamicin-based pTOR-Gateway vectors that expand the possibilities to study oomycete cell biology. In addition, we report that gentamicin-based selection does not alter oomycete virulence. Hence, our findings and resources will enhance the study of oomycete biology as well as plant-oomycete interactions.

## Methods

### Plants and microbial strains and growth conditions

*P. palmivora* growth conditions, maintenance and zoospore production were described elsewhere [25]. *P. infestans* growth conditions, maintenance and zoospore production were described elsewhere [58]. *P. palmivora* strain P16830 (LILI) was isolated from infected oil palm samples harvested in Tumaco Occidental Zone, Colombia [59] and has been obtained from the World Oomycete Genetic Resource collection (https://phytophthora.ucr.edu/). ITS ribosomal sequence can be found under Genbank accession GQ398157. *P. infestans* strain 88,069 (race 1.3.4.7) was isolated from the Netherlands [60] and obtained from The Sainsbury Laboratory, Norwich, UK. Import and maintenance of *P. palmivora* and *P. infestans* are covered by the Department for Environment, Food and Rural Affairs (Defra) plant health licence 114614/208745/4.

*N. benthamiana* is a laboratory cultivar obtained from The Sainsbury Laboratory, Norwich, UK. Its origin dates back to a collection from the Granites site in central Australia which was sent to the United States in 1939 [61]. Growth conditions were described previously [15]. *P. palmivora*, *P. infestans* and *N. benthamiana* were grown and maintained at the Sainsbury Laboratory (SLCU, United Kingdom).

### Plasmid construction

Gentamicin resistance cassette were PCR-amplified from pDONR207 (Invitrogen) vector using the primers GmR_F (5′-ATGTTACGCAGCAGCAACGA-3′) and Hsp70-GmR_IFR (5′-TGGTCGGTCATTTCGAACCCCAGAGTCCCGCTTAGGTGGCGGTACTTGGG-3′). The partial Hsp70 promoter sequence spanning from HpaI restriction site to the beginning of the resistance cassette coding sequence was PCR-amplified from pTORKm43GW using the forward primer Hsp70_IFF (5′-TTATTTAATTTGGTTAACAAATCGGTTTTCGTCGCAAATAGGG-3′) and Hsp70-GmR_R (5′-TCGTTGCTGCTGCGTAACATGCGAAACGGGGCCCTTGTGT-3′). Final amplicons were generated by overlap extension PCR [62] and cloned into a pTORKm43GW by In-Fusion cloning (Clontech, Palo Alto, USA).

### Cleaning up of *Phytophthora* strains

Bacteria growing on *Phytophthora* cultivation plates hamper normal zoospore release and electroporation. To establish axenic *P. palmivora*, we harvested zoospores from a bacteria-contaminated plate and used 10 μL volume of the spore suspension to spot inoculate a new plate containing rifampicin (Rif), cefotaxime (Ctx) and spectinomycin (Spec). After 5-day incubation at 25 °C, an agar plug was taken from fresh *P. palmivora* outgrowth on a Rif/Ctx/Spec plate and subcultured onto a new Rif/Ctx/Spec plate. Mycelia and zoospores produced from these plates were checked for absence of bacterial contamination by inoculation of LB medium with mycelium plugs or zoospore suspension (Supplemental Method). Clean plates were used for further propagation and zoospore electroporation.

### Generation of transgenic *Phytophthora palmivora*

Transgenic *Phytophthora palmivora* were obtained by zoospore electro-transformation using the method from Huitema et al. (2011) with the following modifications: for electroporation, 680 μl of high concentration (> 10^6^ zoospores/ml), high mobility zoospore suspension was mixed with 80 μl of 10× modified Petri’s solution and 40 μl (20–40 μg) of plasmid DNA. Electroporation settings were as follows: voltage 500 V, capacitance 50 μF, resistance 800 Ω. After electroporation, zoospore suspensions were diluted with clarified V8 medium to 5 mL and incubated at 25 °C for 6 h on a rocking shaker. The encysted zoospore suspension was plated on a 15 cm diameter plate with selective medium containing appropriate antibiotics. Transformants were transferred to fresh selective plates up to 10 days after transformation. A detailed procedure can be found in the Supplemental Method.

### Confocal microscopy

Confocal laser scanning microscopy images were acquired with a Leica SP8 laser-scanning confocal microscope equipped with a 25×0.95 numerical aperture (NA) objective (Leica, Wetzlar, Germany). A white-light laser was used for excitation at 477 nm for mTFP1 visualisation, 488 nm for mWasabi visualisation, at 514 nm for mCitrine visualisation and at 543 nm for the visualisation of tdTomato. Fluorescence acquisition was done sequentially. Pictures were analysed with ImageJ software (http://imagej.nih.gov/ij/) and plugin Bio-Formats (https://imagej.net/Bio-Formats).

## Supplementary information

**Additional file 1. Figure S1.** Growth habit of wild-type *P. palmivora* and *P. infestans* strains on several antibiotics. **(A-B)** Representative pictures of 5-day-old *P. palmivora* isolate LILI (accession P16830) grown on V8 **(A)** or 10-day-old *P. infestans* isolate 88,069 grown on RSA **(B)**. Plates were supplemented with 100 mg/L of either carbenicillin, chloramphenicol, cefotaxime, rifampicin, spectinomycin or tetracycline. Scale bar is 30 μm. **Table S1**. Gentamicin-based pTOR-Gateway vectors. Gentamicin resistance conferred by the *aacC1* gene is indicated by the letter G, in addition to the previously described naming conventions. **Supporting protocol.** Step-by-step protocol for electro-transformation of *Phytophthora palmivora* zoospores.

## Data Availability

All data generated or analysed during this study are included in this published article and its supplementary information files. Empty plasmids generated during the current study are available in the Addgene (www.addgene.org/) plasmid repository, under accession numbers 112902 to 112906. Final (recombined) plasmids generated during the current study are available from the corresponding author on reasonable request. Furthermore, the microbial strains *P. palmivora* strain P16830 and *P. infestans* strain 88069 are available.

## Abbreviations

aac(3)-I or aacC1: aminoglycoside 3-N-acetyltransferase I
AAC(3)-I: AMINOGLYCOSIDE 3-N-ACETYLTRANSFERASE I
APH(3′)-II: AMINOGLYCOSIDE 3′-PHOSPHOTRANSFERASE II
ARF: ADP ribosylation factor
G418: geneticin
mTFP1: monomeric Teal Fluorescent Protein 1
nptII: neomycin phosphotransferase
YKDEL: Yellow Fluorescent Protein containing a C-terminal ER retention signal (YFP:KDEL)
