# Supplementary material for: N-acetyltransferase AAC(3)-I confers gentamicin resistance to Phytophthora palmivora and Phytophthora infestans
Source: BMC Microbiol. 2019 Nov 27;19:265. doi: 10.1186/s12866-019-1642-0 (PMC6882347; doi:10.1186/s12866-019-1642-0)
Supplement: Supplementary file 1 — Additional file 1. Figure S1. Growth habit of wild-type P. palmivora and P. infestans strains on several antibiotics. (A-B) Representative pictures of 5-day-old P. palmivora isolate LILI (accession P16830) grown on V8 (A) or 10-day-old P. infestans isolate 88,069 grown on RSA (B). Plates were supplemented with 100 mg/L of either carbenicillin, chloramphenicol, cefotaxime, rifampicin, spectinomycin or tetracycline. Scale bar is 30 μm. Table S1. Gentamicin-based pTOR-Gateway vectors. Gentamicin resistance conferred by the aacC1 gene is indicated by the letter G, in addition to the previously described naming conventions. Supporting protocol. Step-by-step protocol for electro-transformation of Phytophthora palmivora zoospores. [file 12866_2019_1642_MOESM1_ESM.pdf]

Figure S1

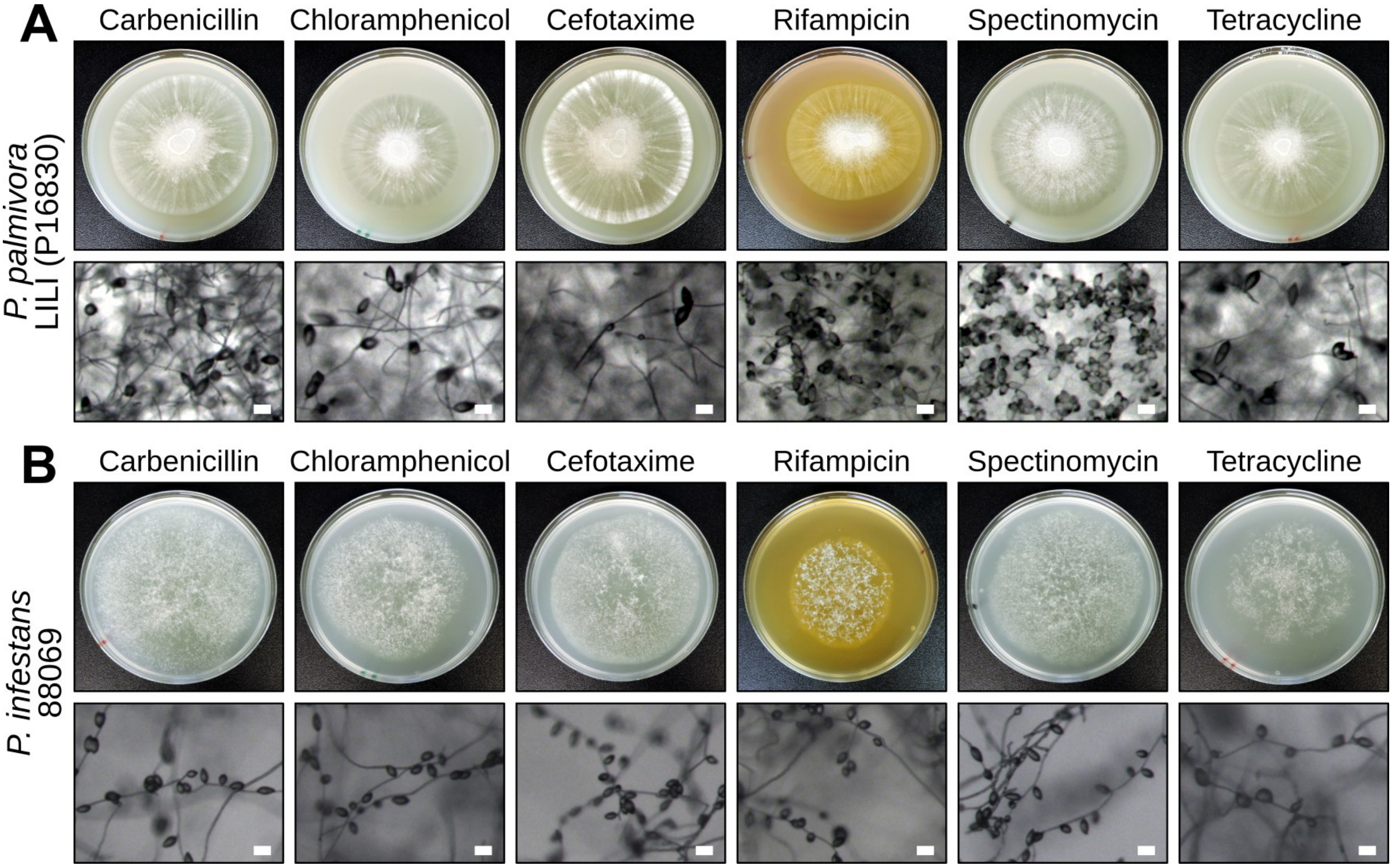

Figure S1. Growth habit of wild-type *P. palmivora* and *P. infestans* strains on several antibiotics. (A-B) Representative pictures of 5-day-old *P. palmivora* isolate LILI (accession P16830) grown on V8 **(A)** or 10-day-old *P. infestans* isolate 88069 grown on RSA **(B)**. Plates were supplemented with 100 mg/L of either carbenicillin, chloramphenicol, cefotaxime, rifampicin, spectinomycin or tetracycline. Scale bar is 30 µm.

Table S1

Plasmid name	Fluorophore	Selection
pTOR**G**m43GW	*None*	Gentamicin
pTOR**GC**m43GW	mTFP1	Gentamicin
pTOR**GF**m43GW	mWasabi	Gentamicin
pTOR**GY**m43GW	mCitrine	Gentamicin
pTOR**GR**m43GW	tdTomato	Gentamicin

Table S1. Gentamicin-based pTOR-Gateway vectors. Gentamicin resistance conferred by the *aacC1* gene is indicated by the letter G, in addition to the previously described naming conventions (Evangelisti et al, 2019).

Supplemental Method

Step-by-step protocol for electro-transformation of *Phytophthora palmivora* zoospores

Evangelisti E[1], Yunusov T[1], Shenhav L[1], Schornack S[1,2]

[1] Sainsbury Laboratory Cambridge University (SLCU), Cambridge, UK.

[2] Author for correspondence: sebastian.schornack@slcu.cam.ac.uk.

Abstract

Electroporation settings for transformation of *Phytophthora capsici* zoospores have been described previously (Huitema *et al.*, 2011). While several aspects of *P. palmivora* transformation resemble those established for *P. capsici*, we substantially modified the original protocol. In particular, we optimized growth conditions and established a new protocol for plasmid preparation. Therefore, we provide below a comprehensive, step-by-step procedure for the establishment and routine maintenance of *P. palmivora* axenic culture, the harvest of high concentration zoospore suspensions from uniformly sporulating plates, as well as electro-transformation and downstream selection of transformants.

Contents

1 Media, equipment and plasmids 1

1.1 Media 1

1.2 Equipment and consumables 2

1.3 Plasmids 2

2 *P. palmivora* axenic culture 2

2.1 Cleanup of *P. palmivora* mycelium 2

2.2 Axenic culture maintenance 2

3 Plasmid preparation 3

4 Transformation 3

4.1 Electroporation 3

4.2 Selection of transformants 4

5 Cryo-preservation 4

1. Media, equipment and plasmids

1.1 Media

Standard recipes for one liter of raw/clarified V8 agar medium are given below. For reference, see Miller PM (1955). *Phytopathology* 45: 461–462.

10% (raw) V8 agar medium. In a beaker, add 100 ml of V8 juice (commercially available) and 1 g of calcium carbonate ($CaCO_3$, Sigma C6763). Mix thoroughly until the calcium carbonate is completely dissolved. Add 0.05 g of β-sitosterol (Sigma S1270) and bring to 1 L with distilled water. Add 15 g of agar (Sigma A1296). Sterilize by autoclaving at 15 psi for 20 min.

10% clarified V8 agar medium. Centrifuge 150 ml of V8 juice containing 1 g of calcium carbonate at 8000 *g* for 5 min. Transfer 100 ml of supernatant to a beaker, add β-sitosterol and agar (see above). Sterilize by autoclaving at 15 psi for 20 min. **Note:** for 10% clarified liquid V8 medium, proceed exactly the same, omitting the agar.

1.2 Equipment and consumables

Required equipment:

- Gene Pulser Xcell Electroporator (Bio-Rad, USA).
- Electroporation cuvettes, 4 mm (VWR 732-1137).
- Rocking shaker.
- Microbiological incubator set at 25°C.
- Microbiological safety cabinet.
- Qubit fluorometer (Thermo Fisher, USA).

Required consumables:

- QIAprep Spin Miniprep Kit (Qiagen, Germany).
- Polypropylene tubes, 15 ml.
- Standard Petri dishes, 90 mm.
- Large Petri dishes, 150 mm.

1.3 Plasmids

Below is a list of plasmids that were successfully transformed with this protocol.

Backbone	Helper cassette	Gateway cassette
pTORKRm43GW	Ham34::tdTomato	*Not recombined* (ccdB)
pTORKFm43GW	Ham34::mWasabi	*Not recombined* (ccdB)
pTORKYm43GW	Ham34::mCitrine	*Not recombined* (ccdB)
pTORKCm43GW	Ham34::mTFP1	*Not recombined* (ccdB)
pTORKm43GW	*None*	Ham34::uidA
pTORKRm43GW	Ham34::tdTomato	UBC2::NLS:mTFP1*
pTORGRm43GW	Ham34::tdTomato	UBC2::NLS:mTFP1
pTORKm43GW	Ham34::mCitrine:CETN2	UBC2::NLS:mTFP1*
pTORKm43GW	CETN2::mScarlet:CETN2	UBC2::NLS:mTFP1*
pTORKm43GW	Ham34::LaminA:mCitrine	UBC2::NLS:mTFP1*
pTORKm43GW	LaminA::LaminA:mCitrine	UBC2::NLS:mTFP1*
pTORGm43GW	*None*	UBC2::Lifeact:mCitrine
pTORKm43GW	Ham34::Lifeact:mCitrine	UBC2::NLS:mTFP1

(*) For reference, see Evangelisti *et al*, 2019

2. *P. palmivora* axenic culture

Electro-transformation of *P. palmivora* zoospores requires production of bacteria-free, high concentration (10^6 zoospores/ml) suspensions of motile zoospores. This is achieved by (1) cleaning-up mycelium using an antibiotic cocktail to reduce bacterial co-cultivation and (2) optimizing growth conditions and mycelium propagation.

2.1 Cleanup of *P. palmivora* mycelium

1. Inoculate a plate of V8 medium[1] containing **rifampicin**, **cefotaxime** and **spectinomycin** at 100 mg/L with a mycelium plug or a droplet of zoospores. Incubate at 25°C[2] for a week.
2. Excise an agar plug **from the growing edge of the mycelium** and transfer to a fresh plate of V8 medium containing the same antibiotics.
3. Incubate at 25°C **in the dark** for 5 days.
4. Incubate **unsealed** (remove any parafilm) for 2 days at 25°C under **constant light** to induce sporangiogenesis[3]. Visual inspection of the plate should then reveal a slightly grainy surface, indicative of sporangia production[4].
5. To harvest zoospores, **incubate for 30 min at 4°C, then add 5 ml of sterile water[5] and monitor zoospore release after 5 min**. Maximum release should occur 5 to 30 min after flooding depending on the strain. Check absence of bacterial contamination on LB medium **(Fig. 1)**.

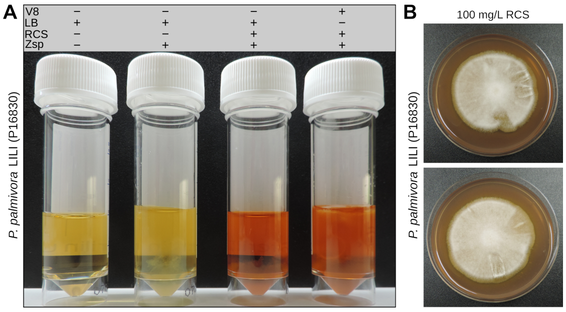

Figure 1. Cleanup of *P. palmivora* mycelium. (A) Representative images of liquid LB or V8 medium containing or not 100 mg/L rifampicin, cefotaxime and spectinomycin, one week after inoculation with *P. palmivora* zoospores. **(B)** Representative pictures of 5-day-old *P. palmivora* isolate LILI (accession P16830) grown on V8 supplemented with 100 mg/L of rifampicin, cefotaxime and spectinomycin. Abbreviations: RCS: rifampicin, cefotaxime and spectinomycin; Zsp: zoospores; V8: 10% clarified liquid V8 medium; LB: Luria-Bertani broth.

2.2 Axenic culture maintenance

1. Inoculate a plate of V8 medium with **4 equally spread mycelium plugs**, or **1 ml of zoospore**

[1] Other *Phytophthora* species may grow better on rye-sucrose agar (RSA) medium.

[2] Incubation temperature should be adapted for other *Phytophthora* species.

[3] Unsealed plates start drying, which promotes zoospore release from sporangia.

[4] In some cases, plates look thick and fluffy. In those plates, sporangia are overlaid by mycelium.

[5] If water is repelled, tap the plate surface with a pipette tip to let it soak through.

suspension. In our hands, the latter gives more sustained zoospore yield over time.

2. Follow steps 3 to 5 from section 2.1 (see above).

3. Plasmid preparation

We achieved successful *P. palmivora* transformation using at least 20 μg of plasmid DNA. Plasmid can be prepared using maxiprep kits from various manufacturers. Below is an alternative protocol using a plasmid miniprep kit (Qiagen, Germany) that we routinely use in the lab. Critical steps are indicated in **bold**.

1. Inoculate 100 ml of LB medium with a single *Escherichia coli* colony.
2. Incubate overnight at 37°C under vigorous shaking.
3. Centrifuge the overnight culture and resuspend the pellet in **4 ml** of buffer P1.
4. Add **4 ml** of buffer P2, mix and incubate for **5 min** at room temperature.
5. Add **4 ml** of buffer N3, **mix thoroughly** and distribute to 10 microfuge tubes.
6. Centrifuge at full speed ($> 20\,000$ g) for 2 min.
7. Transfer the supernatant to spin columns.
8. Centrifuge at full speed for 30 s.
9. Discard the flow-through. Add 700 μl of buffer PE.
10. Centrifuge at full speed for 30 s.
11. Discard the flow-through. Transfer columns to new collection tubes.
12. Centrifuge at full speed for 1 min.
13. Apply **80 μl** of nuclease-free water to 2 columns.
14. Centrifuge at full speed for **1 min**.
15. **Elute 2 more columns with flow-through.**
16. Repeat steps 14 and 15 with remaining columns. Flow-through volume may reach 30 µl/column.
17. **Pool the final eluted fractions**.

Note: Qiagen spin miniprep columns have a maximum binding capacity of 20 μg plasmid DNA. Quantify DNA with a Qubit fluorometer (Thermo Fisher, USA) using broad range (BR) buffer.

4. Transformation

P. palmivora electro-transformation is achieved using a Gene Pulser Xcell Electroporator (Bio-Rad, USA). Other electroporation systems can be used, but electrical settings may need to be adjusted.

4.1 Electroporation

1. Set up the electroporator as follows: use **exponential decay** mode, set voltage to **500 V**, capacitance to **50 μF**, resistance to **800 ohms** and cuvette size to **4 mm**.
2. Harvest *P. palmivora* zoospores (see section 2.1, step 5). Check the suspension for zoospore density[6] and motility. In our hands, fast-moving zoospores yield more transformants.
3. In a 4-ml electroporation cuvette, mix **80 μl** of 10$\times$ modified Petri's solution, **20-40 μg** of plasmid DNA and bring to a final volume of **800 μl** with zoospore suspension. **Zoospores** are sensitive to physical stimuli and **should be handled gently**, especially when pipetting.
4. Apply one pulse per cuvette **without delay**.
5. Immediately transfer the electroporation mix to a 15 ml polypropylene tube, and **bring to 5 ml with liquid V8 medium.**
6. Incubate for 4 to 6 hours at 25°C on a **rocking shaker**. Germinating cysts should aggregate to mycelium clumps within 2 to 4 hours, depending on zoospore concentration and growth speed.
7. Pour the mycelium clumps on a 15-cm plate containing clarified V8 agar medium supplemented with appropriate antibiotics. Spread by tilting.
8. Seal and incubate at 25°C in the dark for **10 days**. Typical regenerants are shown in **Fig. 2**.

Modified Petri's solution, 10$\times$ concentrate:

- 2.5 mM $CaCl_2$,
- 10 mM $MgSO_4$,
- 10 mM KH_2PO_4,
- 8 mM KCl.

Filter-sterilize and store at 4°C.

[6] Precise counting with a haemocytometer is not required.

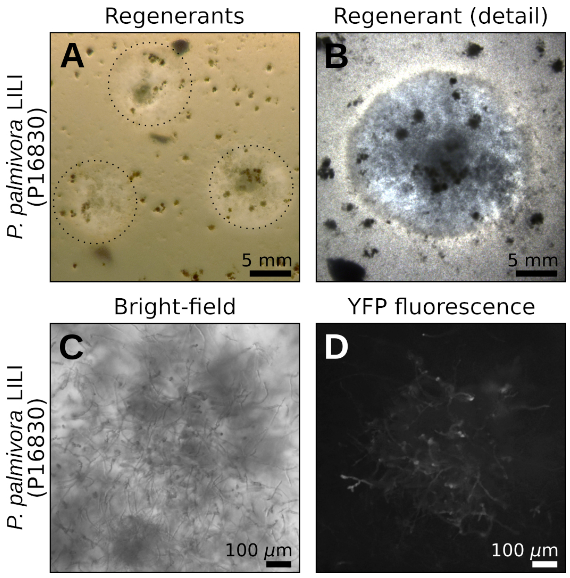

Figure 2. Growth habit of *P. palmivora* regenerants. (A) Representative images of regenerants on a V8 medium plate containing 100 mg/L geneticin (G418). Dark spots indicate mycelium clumps. **(B)** Detailed view of a single regenerant. **(C-D)** Representative images of hyphae from a validated transformant expressing a Lifeact:mCitrine actin fluorescent reporter, under bright-field illumination **(C)** and YFP fluorescence **(D)**.

4.2 Selection of transformants

Screening by fluorescence

Regenerants obtained after transformation with a pTORKm43GW or pTORGm43GW vector carrying a Ham34-promoter-driven fluorophore (mTFP1, mWasabi, mCitrine or tdTomato) can be screened for hyphal fluorescence. **Regenerants showing fluorescence should be further screened for expression of the construct inserted in the Gateway cassette**. Indeed, uncoupled expression of the two cassettes has been reported (Evangelisti *et al*, 2019).

Screening by PCR

When fluorescence is not available, presence of the transgene should be assessed by PCR. *P. palmivora* genomic DNA can be extracted from mycelium or zoospores using standard STES (Möller *et al*, 1992) or CTAB (Kim *et al*, 1990) protocols.

Note: Transgene expression level can vary within a single colony mycelium and also between independent transformants. We therefore do not recommend the establishment of monozoosporic lines from these transformants.

5. Cryo-preservation

Long-term storage of *P. palmivora* mycelium can be achieved through cryo-preservation in liquid nitrogen. For comparison of various cryo-preservation protocols, see Houseknecht *et al*, 2012.

1. Excise up to 6 agar plugs (1 cm × 1 cm) from a **10-day-old plate** containing sporangia.
2. Transfer the agar plugs to a cryotube (screw lid).
3. **Immerse the agar plugs** in filter-sterilized 5% dimethyl sulfoxide (DMSO) aqueous solution.
4. **Incubate at −80°C** to allow for slow freezing (a −1°C/min temperature decrease rate can be precisely controlled using a freezing container). Keep at least **24 hours**. **Note:** samples can be kept at −80°C for several months.
5. Transfer to a liquid nitrogen storage tank. The strain should be stable for years.
6. To revive a strain, **incubate a frozen cryotube in a 40°C water bath for to 3 min**.
7. **Transfer the agar plugs on a fresh V8 plate** (containing appropriate antibiotics), after soaking up excess DMSO on a sterile Whatman paper.

Literature cited

Evangelisti E, Shenhav L, Yunusov T, Le Naour-Vernet M, Rink P, Schornack S (2019). Centrin-anchored hydrodynamic shape changes underpin active nuclear rerouting in branched hyphae of an oomycete pathogen. *bioRxiv* doi:10.1101/652255.

Houseknecht JL, Suh SO, Zhou JJ (2012). Viability of fastidious *Phytophthora* following different cryopreservation treatments. *Fungal Biol.* **116**(10):1081–1089.

Huitema E, Smoker M, Kamoun S (2011). A straightforward protocol for electro-transformation of *Phytophthora capsici* zoospores. *Methods Mol. Biol.* 712: 129–135.

Kim WK, Mauthe E, Hausner G, Klassen GR (1990). Isolation of high molecular weight DNA and double-stranded RNAs from fungi. *Can. J. Bot.* **68**(9): 1898–1902.

Miller PM (1955). V-8 juice agar as a general-purpose medium for fungi and bacteria.*Phytopathology* 45: 461–462.

Möller EM, Bahnweg G, Sandermann H, Geiger HH (1992). A simple and efficient protocol for isolation of high molecular weight DNA from filamentous fungi, fruit bodies, and infected plant tissues. *Nucl. Acid Res.* **20**(22): 6115–6116.
